# Abdominal Wall Perivascular Epithelioid Cell Tumor Mimicking an Intra-Abdominal Tumor: A Case Report

**DOI:** 10.70352/scrj.cr.25-0826

**Published:** 2026-03-19

**Authors:** Saki Kubota, Yoshiaki Fujimoto, Takuya Honboh, Kosuke Hirose, Taichi Nagano, Huanlin Wang, Fumihiko Hirai, Noboru Harada, Seiya Kato, Noriaki Sadanaga, Tomoharu Yoshizumi

**Affiliations:** 1 Department of Surgery, Saiseikai Fukuoka General Hospital, Fukuoka, Fukuoka, Japan; 2 Department of Pathology, Saiseikai Fukuoka General Hospital, Fukuoka, Fukuoka, Japan; 3 Department of Surgery and Science, Graduate School of Medical Sciences, Kyushu University, Fukuoka, Fukuoka, Japan

**Keywords:** abdominal wall tumor, PEComa, perivascular epithelioid cell tumor, laparoscopic surgery, soft tissue neoplasm, case report

## Abstract

**INTRODUCTION:**

Perivascular epithelioid cell tumors (PEComas) are rare mesenchymal neoplasms; those arising from the abdominal wall are exceptionally uncommon. Because imaging findings are nonspecific, an abdominal wall PEComa may be mistaken for an intra-abdominal tumor, leading to diagnostic uncertainty and challenges in surgical planning.

**CASE PRESENTATION:**

A 25-year-old woman was referred to our hospital after a screening of the upper gastrointestinal series revealed extrinsic compression of the stomach. Cross-sectional imaging demonstrated a well-circumscribed midline mass with early enhancement and calcifications, which was initially suspected to be intra-abdominal in origin. Because the precise anatomical origin of the lesion remained unclear and malignancy could not be excluded, a laparoscopic first-look approach was employed. Intraoperative inspection confirmed that the tumor originated from the subperitoneal abdominal wall, without intraperitoneal involvement. The lesion was resected en bloc with negative margins through a limited incision. The histopathological and immunohistochemical findings were consistent with those of a PEComa with malignant potential. The postoperative course was uneventful, and no recurrence was observed during follow-up.

**CONCLUSIONS:**

Abdominal wall PEComa is an extremely rare entity that can closely mimic an intra-abdominal tumor on imaging studies. When the anatomical origin of a midline mass cannot be clearly determined preoperatively, a laparoscopic first-look approach is a useful strategy for clarifying the tumor’s origin and guiding safe surgical management. Long-term follow-up is warranted because of the malignant potential of PEComas.

## Abbreviations

ADC
: apparent diffusion coefficient
DWI
: diffusion-weighted imaging
HPF
: high-power field
PEComa
: perivascular epithelioid cell tumor

## INTRODUCTION

PEComas are rare mesenchymal neoplasms composed of distinctive perivascular epithelioid cells that co-express melanocytic and smooth muscle markers, and they can arise at various anatomical sites.^1)^ Although PEComas have been reported in organs such as the liver, uterus, and soft tissues, their overall incidence is extremely low. Abdominal wall PEComas are exceptionally rare, with only isolated case reports available.^1,2)^

The preoperative diagnosis of abdominal wall PEComas is challenging because the imaging findings are nonspecific and may closely resemble those of more common soft-tissue tumors.^2)^ In particular, lesions located along the midline of the abdominal wall can mimic intra-abdominal tumors on radiographic and cross-sectional imaging, potentially leading to diagnostic uncertainty and an unexpected clinical course regarding the true site of origin.^2,3)^

Accurate identification of the tumor origin is essential for appropriate surgical planning, especially when malignancy cannot be excluded. We report a case of abdominal wall PEComa which was initially suspected to be an intra-abdominal tumor. A diagnostic laparoscopy (first-look approach) was successfully employed to determine the tumor origin and achieve a safe, margin-negative resection. This case highlights the diagnostic pitfalls associated with this rare entity, and demonstrates the practical value of laparoscopy as a problem-solving strategy for anatomically ambiguous tumors.

## CASE PRESENTATION

### Patient background and presentation

A 25-year-old woman with a BMI of 17.8 and no significant medical or surgical history was referred to our department after an upper gastrointestinal screening radiography showed extrinsic compression of the greater curvature from the gastric angle to the antrum. She had no history of hormonal therapy, pregnancy, or features suggestive of tuberous sclerosis complex or lymphangioleiomyomatosis. She was asymptomatic and had normal physical and laboratory findings.

### Preoperative imaging

Upper gastrointestinal series demonstrated a smooth extrinsic impression on the greater curvature of the distal stomach, suggestive of a mass effect from outside the gastric wall.

**Fig. 1:**
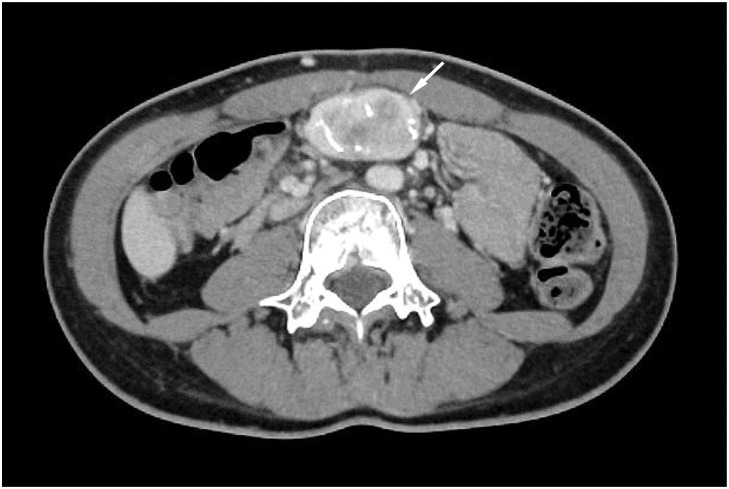
Contrast-enhanced CT of the abdominal wall mass. Axial contrast-enhanced CT demonstrates a 45-mm well-circumscribed solid mass with coarse internal calcifications located between the supraumbilical and umbilical abdominal wall (arrow). The lesion abuts the posterior aspect of the abdominal wall without any evidence of intraperitoneal extension.

Contrast-enhanced CT revealed a 45-mm well-circumscribed solid lesion with coarse internal calcifications located along the midline of the abdominal wall between the supraumbilical and umbilical levels (**Fig. 1**). The lesion appeared to be immediately deep to the anterior abdominal wall; however, the distinction between an abdominal wall and an intra-abdominal origin remained unclear. No intra-abdominal or intrapelvic lesions were identified.

**Fig. 2:**
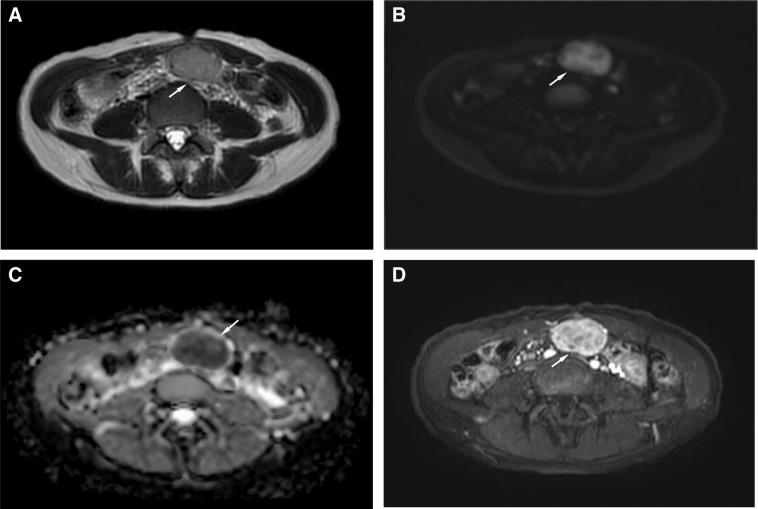
MRI shows T2 hyperintensity, diffusion restriction, and early enhancement. (**A**) T2-weighted imaging shows a well-defined, hyperintense mass (arrow). (**B**) Diffusion-weighted imaging demonstrates high signal intensity within the lesion (arrow). (**C**) The corresponding apparent diffusion coefficient (ADC) map shows low signal intensity, consistent with diffusion restriction (arrow). (**D**) Dynamic contrast-enhanced imaging reveals early enhancement of the tumor, suggestive of a hypervascular soft-tissue neoplasm (arrow).

MRI demonstrated that the mass was isointense on T1-weighted images and hyperintense on T2-weighted images, with high signal intensity on DWI and low ADC values. Dynamic contrast-enhanced sequences demonstrated early enhancement (**Fig. 2**). No macroscopic fat component was identified, and the findings were considered compatible with a malignant soft tissue tumor such as sarcoma. PET-CT was not performed.

Based on these findings, the preoperative differential diagnoses included desmoid-type fibromatosis, leiomyoma, and soft-tissue sarcoma. The exact origin (abdominal wall vs. intra-abdominal) remained ambiguous.

### Surgical procedure

Because of the possibility of malignancy and uncertainty regarding the tumor origin, we elected to perform a diagnostic laparoscopy to assess the relationship between the mass and the peritoneal cavity.

**Fig. 3:**
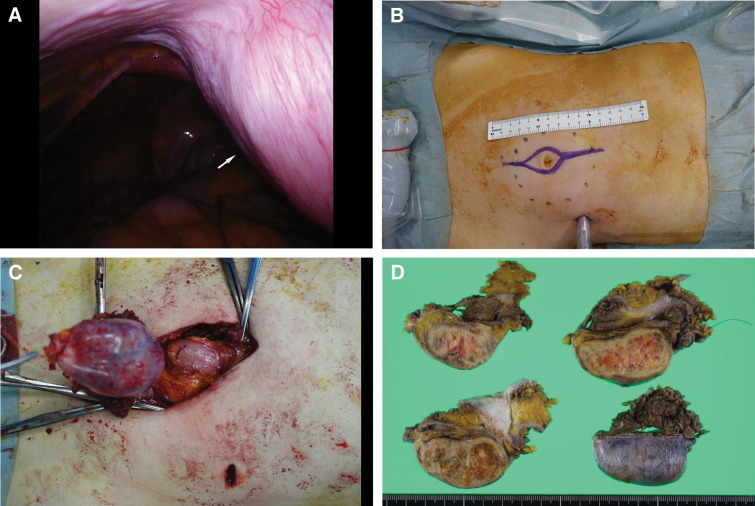
Intraoperative findings and the surgical approach. (**A**) Diagnostic laparoscopy (first-look approach) from a left upper abdominal port reveals a bulging subperitoneal mass integrated with the abdominal wall in the umbilical region (arrow), with no intraperitoneal involvement. (**B**) External operative field showing the planned spindle-shaped skin incision to achieve adequate surgical margins. (**C**) En bloc resection of the mass through the abdominal wall while avoiding direct tumor manipulation and preserving a safe dissection plane. (**D**) Gross appearance of the resected specimen demonstrates en bloc removal of the abdominal wall tumor with adequate surrounding tissue.

A 12-mm camera port was placed in the left upper abdomen and diagnostic laparoscopy was performed. Intraoperatively, a bulging subperitoneal mass was observed in the umbilical region, which was integrated into the abdominal wall and covered by an intact peritoneum. No intraperitoneal extensions or associated lesions were observed (**Fig. 3**).

After confirming that the tumor originated from the abdominal wall, the peritoneum was incised with an adequate margin around the mass. The urachal remnant and round ligament were ligated and divided to secure a safe, en bloc resection plane. Special care was taken to avoid direct grasping or compressing the tumor in order to prevent capsular disruption.

The lesion was then removed en bloc through a 10 × 4 cm spindle-shaped skin incision over the umbilical region. The resulting fascial defect was closed using a tension-reduction technique by performing relaxing incisions on the anterior rectus sheath, followed by primary suture repair without mesh reinforcement. No intraoperative complications occurred.

### Pathological findings

Gross examination revealed a well-circumscribed subperitoneal mass measuring 60 × 35 × 30 mm. The cut surface was solid and tan, with focal sclerosis and no obvious hemorrhage or necrosis.

**Fig. 4:**
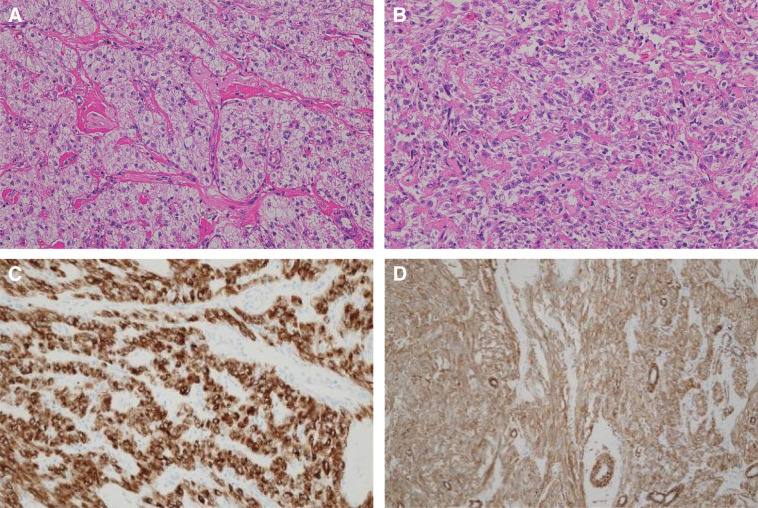
Histopathological and immunohistochemical findings of the tumor. (**A**) High-power hematoxylin and eosin staining shows a well-circumscribed tumor composed of peripheral nests of clear epithelioid cells with granular cytoplasm. (**B**) High-power view demonstrates fascicular proliferation of spindle-shaped cells in the central portion of the tumor. (**C**) Immunohistochemistry shows diffuse cytoplasmic positivity for HMB-45. (**D**) Tumor cells are also positive for α-smooth muscle actin, supporting the diagnosis of a perivascular epithelioid cell tumor.

Histologically, the peripheral portion of the tumor was composed of nests and sheets of clear epithelioid cells with a granular cytoplasm, whereas the central portion exhibited fascicular proliferation of spindle-shaped cells (**Fig. 4A** and **4B**). Moderate nuclear atypia was observed. The mitotic count was 1–2 per 10 HPF and tumor necrosis was not evident.

Immunohistochemically, the tumor cells were positive for HMB-45, α-smooth muscle actin, and calponin, and weakly positive for Melan-A and S-100. The cells were negative for desmin and c-kit (**Fig. 4C** and **4D**). The Ki-67 proliferation index was approximately 1%. These findings were consistent with the diagnosis of PEComa. The surgical resection margins were histologically negative.

According to the widely used criteria proposed by Folpe et al., tumor size >5 cm and increased mitotic activity are associated with malignant behavior.^1)^ Our case fulfilled both these criteria and was therefore classified as PEComa with malignant potential.

### Postoperative course and follow-up

The postoperative course was uneventful. The patient resumed oral intake on POD 2 and was discharged on POD 9. Follow-up CT at regular intervals demonstrated no local recurrence or distant metastasis within 14 months after the surgery.

## DISCUSSION

PEComas are rare mesenchymal neoplasms, and their occurrence in the abdominal wall is exceptionally uncommon.^1,3)^ Most PEComas arise in the retroperitoneum, uterus, gastrointestinal tract, or visceral soft tissues, whereas abdominal wall involvement has been described only sporadically.^1)^ Because of this extreme rarity, the clinical characteristics and optimal management strategies for abdominal wall PEComas have not been well established, and each additional case contributes valuable information to the existing literature.

The preoperative diagnosis of PEComa is challenging because radiological findings are nonspecific and frequently overlap with those of more common benign and malignant soft-tissue tumors.^2,3)^ In the present case, the tumor was initially suspected to be intra-abdominal because it caused extrinsic compression of the stomach and demonstrated early contrast enhancement with calcification on imaging. This atypical presentation resulted in diagnostic uncertainty regarding the true anatomical origin of the lesion. Midline abdominal wall tumors, particularly those located in the subperitoneal space, may closely mimic intra-abdominal masses, leading to an unexpected clinical course and potentially inappropriate surgical planning, if this possibility is not considered.

Accurate identification of the tumor origin is crucial for appropriate surgical decision-making, especially when malignancy cannot be excluded. When imaging studies fail to clearly distinguish between abdominal wall and intra-abdominal tumors, proceeding directly to open exploration may result in unnecessary invasiveness or suboptimal incision placement. In this context, a laparoscopic first-look approach offers a minimally invasive means of directly assessing the peritoneal cavity and clarifying the tumor origin. In the present case, laparoscopy confirmed that the lesion originated from the abdominal wall without intraperitoneal involvement, enabling precise incision planning and safe en bloc resection with negative margins, while minimizing surgical trauma. This experience suggests that laparoscopy may serve as an effective problem-solving tool in cases of anatomically ambiguous tumors.

Histopathologically, the tumor demonstrated a mitotic count of 1–2 per 10 high-power fields, which corresponds to approximately 5–10 mitoses per 50 high-power fields and therefore exceeds the threshold proposed in the Folpe criteria, supporting malignant potential. In this context, the widely used criteria proposed by Folpe et al. define worrisome features, including tumor size >5 cm, infiltrative growth, high nuclear grade and cellularity, mitotic activity >1 per 50 high-power fields, coagulative necrosis, and vascular invasion; tumors meeting two or more of these features are considered malignant, whereas tumors with only one worrisome feature are often regarded as having uncertain/malignant potential.^1)^ Given the rarity of PEComas—particularly those arising from the abdominal wall—robust site-specific prognostic data and standardized surveillance protocols remain limited. Nevertheless, delayed local recurrence and distant metastasis have been reported, supporting the rationale for long-term imaging-based follow-up even after complete resection. In clinical practice, a reasonable surveillance approach is periodic cross-sectional imaging for several years (e.g., every 6–12 months initially, then annually), tailored to the patient’s risk features and institutional practice.

The biological behavior of PEComas ranges from benign to malignant, and histopathological criteria, such as tumor size and mitotic activity, are important predictors of aggressive behavior. In the present case, the tumor exceeded 5 cm in size and showed mitotic activity, fulfilling the criteria of malignant potential. Although no recurrence has been observed during follow-up, delayed recurrence and metastasis have been reported in PEComa, underscoring the need for careful long-term surveillance even after complete surgical resection.^1,4)^

Overall, this case highlights the extreme rarity of abdominal wall PEComas, the possibility of an unexpected diagnostic course because of misleading imaging findings, and the practical value of a laparoscopic first-look approach in achieving an accurate diagnosis and safe surgical management. Awareness of these features may help surgeons to appropriately evaluate and manage similar cases in future clinical practice.

## CONCLUSIONS

Abdominal wall PEComa is an extremely rare entity that can closely mimic an intra-abdominal tumor on imaging studies, leading to diagnostic uncertainty and an unexpected clinical course. When the anatomical origin of a midline mass cannot be clearly determined preoperatively, a laparoscopic first-look approach provides a minimally invasive and effective means of clarifying the tumor origin and guiding appropriate surgical planning. This strategy enabled safe margin-negative resection in the present case and may be valuable in the management of other anatomically ambiguous abdominal wall tumors. Careful long-term follow-up is warranted in view of the malignant potential of PEComa.

## DECLARATIONS

### Availability of data and materials

The datasets supporting the conclusions of this study are included within this article.
